# The complete mitochondrial genome of *Lycodon liuchengchaoi* (squamata: colubridae)

**DOI:** 10.1080/23802359.2016.1219639

**Published:** 2016-09-04

**Authors:** Chencheng Wang, Chenling Zhang, Lifu Qian, Baowei Zhang, Hui Wang

**Affiliations:** a School of Life Sciences, Anhui University, Hefei, Anhui, People’s Republic of China;; b Faculty of Life Science and Chemical Engineering, Jiangsu Second Normal University, Nanjiang Jiangsu, People’s Republic of China

**Keywords:** *Lycodon liuchengchaoi*, mitochondrial genome, phylogenetic tree

## Abstract

*Lycodon liuchengchaoi* is a new species discovered in recent years which is widely distribute in Anhui, Hubei and Sichuan Province. In this study, we determined the complete mitochondrial genome of *L. liuchengchaoi*. The result shows that the complete mitogenome of *L. liuchengchaoi* is 17,171bp. It is similar with the typical mtDNA of Serpentes, which contains 13 protein-coding genes, 2 rRNA genes, 22 tRNA genes, 2 control regions, and a stem-loop region. The phylogenetic tree, contains 17 Serpentiforms species, is divided into two clades which correspond to six genera in Colubridae. The *L. liuchengchaoi* which appeared into Clade A, clustered within *Lycodon*.

The *Lycodon liuchengchaoi* is a species of Colubridae family (Zhang et al. 2011). *Lycodon liuchengchaoi*, once misidentified as *L. fasciatus* because of their similarity in shape (Lei et al. 2014; Ding et al. 2015). However, in 2011, it was identified as a new species after the careful examination of these specimens that was collected from Sichuang Province (Zhang et al. 2011), the researchers noticed that they can be distinguished from *L. fasciatus* by several morphological characters, such as the anal plate and the large number and yellow colour of the rings around the body (Vogel et al., 2010; Zhang et al. 2011). In the following research, some new records about *L. liuchengchaoi* were reported in Anhui and Hubei Province (Zhang et al. 2015). In the present study, the *L. liuchengchaoi* sample was collected from Yaoluoping, Anqing of Anhui province, China (N 30°57′57.06″, E 116°04′04.96″). Now, the specimen was deposited in the laboratory of Evolution and Ecology, School of Life Sciences, Anhui University.

We sheared the muscle tissue to extract the whole genomic DNA using a standard proteinase-K/phenol–chloroform protocol (Sambrook & Russell 2006). The entire mitogenome was amplified using 16 pairs of primers by PCR. Here, we sequenced the complete mitochondrial genome of *L. liuchengchaoi* and submitted it to GenBank (accession no. KX553922). Phylogenetic relationship of 17 Colubridae species were analyzed with the Bayesian inference (BI) method using the MrBayes version 3.1.2 software (University of Rochester, NY) (Huelsenbeck & Ronquist 2001) based on nucleotide sequences of Cyt *b* gene, selecting *Python molurus* as an outgroup. In this process, the best-fitting nucleotide substitution model (GTR + I + G) was selected via MrModeltest version 2.1 (Uppsala University, SWE) (Nylander et al. 2004); the four independent Markov’s chain runs for 1,000,000 metropolis-coupled Monte Carlo (MCMC) generations, sampling every 1000 generations. When the average standard deviation of split frequencies reached a value less than 0.01, the first 1000 trees were discarded as ‘burn-in’ and the remaining trees were used to calculate the Bayesian posterior probabilities (Pan et al. 2015).

The complete mitogenome sequence of *L. liuchengchaoi* is 17,171 bp in length, and the gene order was identical to *L. semicarinatum*, including 13 protein-coding genes, 22 tRNA genes, 2 rRNA genes, 2 control regions, and a stem-loop region. The base composition of the mitogenome was 33.8% A, 28.2% C, 13.0% G, and 25.0% T. The *ND6* subunit gene, stem-loop, and eight tRNA genes (*tRNA^Pro^*, *tRNA^Gln^*, *tRNA^Ala^*, *tRNA^Asn^*, *tRNA^Cys^*, *tRNA^Tyr^*, *tRNA^Ser^*^(UCN)^, and *tRNA^Glu^*) were encoded on the L-strand, the others were encoded on the H-strand.

According to the Bayesian analysis, the phylogenetic tree divided the species into two clades, these relationships received well support (Figure 1). The Clade A contains *Lycodon*, *Elaphe*, *Coluber*, *Calamaria*, and *Amphiesma*. The *Pareas* presents in the Clade B. In the phylogenetic tree, the species of sample in this study named as *L. liuchengchaoi* (1) cluster with the sequence of *L. liuchengchaoi* (Zhang et al. 2015), which appeared in the genus of *Lycodon*, Clade A. Our study of *L. liuchengchaoi* can provide a useful database for analyzing the classification and status in Colubridae.

**Figure 1.**
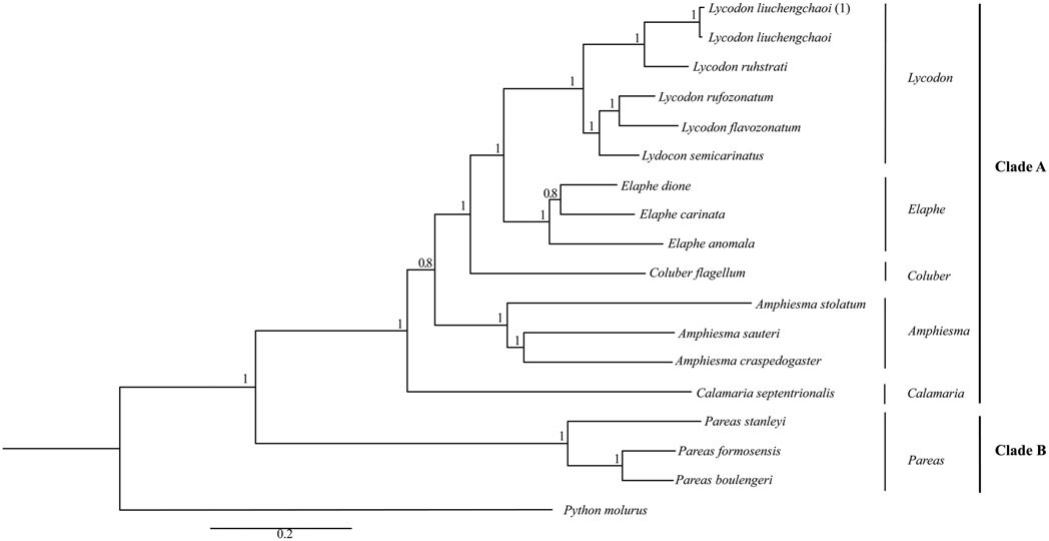
The phylogenetic relationships analysis among the species in Colubridae is based on the Cyt b gene using Bayesian inference (BI). The percentages of Bayesian’s posterior probabilities (BPPs) show as the numbers at each node. GenBank accession numbers Cyt b are: *L. liuchengchaoi* (KP898899), *L. ruhstrati* (NC029153), *L. rufozonatum* (NC028730), *L. flavozonatum* (NC028730), *L. semicarinatus* (NC001945), *E. dione* (HQ830257), *E. carinata* (KF669252), *E. anomala* (KP900218), *C. flagellum* (KM403637), *A. stolatum* (KJ685711), *A. crapedogaster* (GQ281178), *A. sauteri* (AF402905), *C. septentrionalis* (KR814699), *P. stanleyi* (JN230704), *P. formosensis* (JF827693), *P. boulengeri* (JF827683) and *P. molurus* (AY099983).
